# Highly tough, crack‐resistant and self‐healable piezo‐ionic skin enabled by dynamic hard domains with mechanosensitive ionic channel

**DOI:** 10.1002/smo.20240008

**Published:** 2024-08-19

**Authors:** XueBin Wang, Tong Liu, FuYao Sun, Jingyi Zhang, BoWen Yao, JianHua Xu, JiaJun Fu

**Affiliations:** ^1^ School of Chemistry and Chemical Engineering Nanjing University of Science and Technology Nanjing China

**Keywords:** crack resistance, piezo‐ionic dynamics, self‐healing

## Abstract

Robust and reliable piezo‐ionic materials that are both crack resistant and self‐healable like biological skin hold great promise for applications inflexible electronics and intelligent systems with prolonged service lives. However, such a combination of high toughness, superior crack resistance, autonomous self‐healing and effective control of ion dynamics is rarely seen in artificial iontronic skin because these features are seemingly incompatible in materials design. Here, we resolve this perennial mismatch through a molecularly engineered strategy of implanting carboxyl‐functionalized groups into the dynamic hard domain structure of synthesized poly(urethane‐urea). This design provides an ultra‐high fracture energy of 211.27 kJ m^−2^ that is over 123.54 times that of tough human skin, while maintaining skin‐like stretchability, elasticity, and autonomous self‐healing with a 96.40% healing efficiency. Moreover, the carboxyl anion group allows the dynamic confinement of ionic fluids though electrostatic interaction, thereby ensuring a remarkable pressure sensitivity of 7.03 kPa^−1^ for the tactile sensors. As such, we successfully demonstrated the enormous potential ability of this skin‐like piezo‐ionic sensor for biomedical monitoring and robotic item identification, which indicates promising future uses in flexible electronics and human–machine interactions.

## Graphical abstract

We propose a biomimetic strategy of introducing carboxyl anion into the hard segment of self‐healing poly(urethane‐urea), assembling a novel dynamic hard domain with dynamic ion‐confining ability, which successfully surpassed the notorious conflict between autonomous self‐healing, high toughness, crack‐resistance and piezo‐ionic dynamics in artificial iontronic skin.

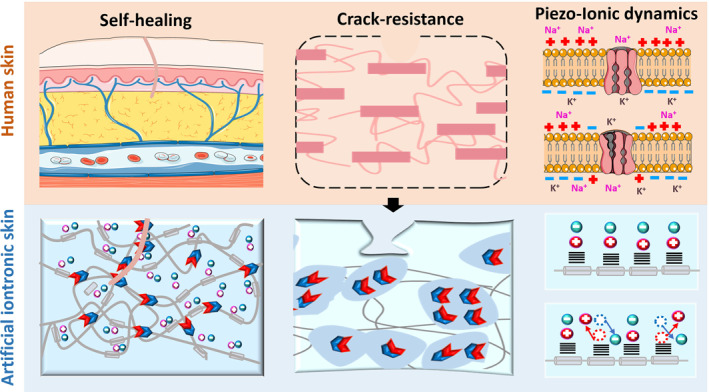

## 1 INTRODUCTION

**FIGURE 1:**
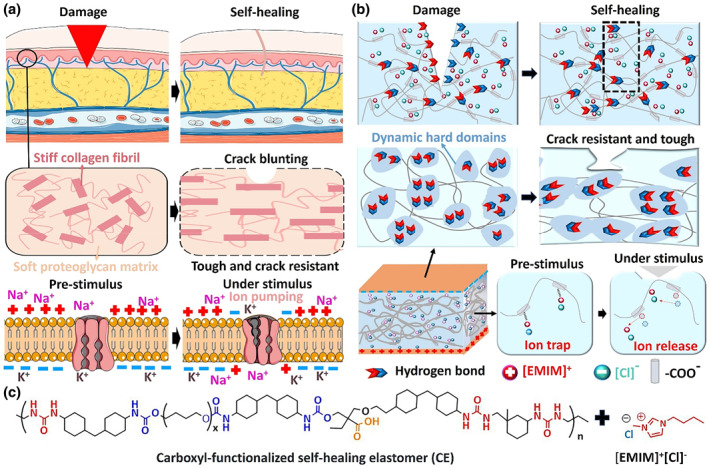
Conceptual design principle of CPIE. (a) Schematic diagram depicting the self‐healing process and tactile sensation generation of human skin. The collagen fibers and elastic fibers in the dermis staggered together to passivate the cracks, imparting the human skin with excellent crack resistance. The human skin can self‐heal with the assistance of blood cells and platelets when damaged. It also detects external stimulus through the mechanoreceptor composed of tethered ion channels. (b) Schematic illustration of the autonomous self‐healing of CPIE‐based e‐skin, an elaborate molecular design strategy using staggered dynamic hard domains enhances the structure's resistance to crack propagation and the piezoelectric ion dynamics simulating the ion trap and release of carrier proteins. (c) Molecular engineering of the design rule of CPIE.

Human skin not only has a mechanosensory system capable of detecting pressure, strain and torsion based on iontronics, but also show spontaneous healing to ensure the integrity and functional stability after damage (Figure 1a).[^1^, ^2^] Inspired by such a combined sensory and self‐healing structure, several bionic skins developed from iontronic materials have been reported, which take ions as charge carries (that is similar to nerve signal conduction), ensuring high spatial resolution,[^3^, ^4^] exceptional noise immunity,[^5^, ^6^] and superior sensitivity to both dynamic and static stimuli.[^6^, ^7^] Meanwhile, their remarkable self‐healing ability facilitate the restoration of mechanical damage and broken functionalities to extent operational longevity of a device.[^8^, ^9^, ^10^] Generally, the bionic skins are divided into four types based on different physical stimuli, that is, piezoresistive, capacitive, piezoelectric and triboelectric bionic skins.^[11]^ Among them, capacitive bionic skins, which exhibit high sensitivity, fast response time, and low power consumption, have been widely investigated.[^12^, ^13^, ^14^] One typical example was demonstrated by Kim and co‐workers, where they introduced Cl‐functionalized groups into a polyurethane matrix containing ionic liquid (IL), realizing an autonomous self‐healing and excellent pressure sensitivity in a capacitive bionic skin.^[15]^ However, an enormous challenge arises due to their poor crack resistance, resulting in reduction or loss of the device operation reliability. The simultaneous achievements of high fracture toughness and autonomous self‐healing in an iontronic material become crucial for long‐term applications.

Conventionally, traditional self‐healing iontronic materials are crosslinked by weak non‐covalent bonds such as hydrogen bonds,[^16^, ^17^, ^18^, ^19^, ^20^, ^21^] metal coordination,[^22^, ^23^, ^24^, ^25^, ^26^] and ionic interactions,[^16^, ^27^] which are capable of reversibly recombining at crack interfaces to ensure the autonomous self‐healing performance owning to their high dynamics and weak bond strength.[^28^, ^29^] Nevertheless, the threshold fracture toughness of a covalent polymer scales with the chemical energy per covalent bond, that is, the stronger the bond strength, the tougher the polymer becomes in theory.^[30]^ Consequently, the autonomous self‐healing properties of iontronic materials often compromise the high toughness in principle.[^31^, ^32^, ^33^] To address this concern, previous studies have assembled incompatible supramolecular aggregates to form nano/microscale phase separation structures, realizing toughening without sacrificing autonomous self‐healing.[^34^, ^35^, ^36^] For example, our group recently introduced a design concept of “dynamic hard domains” assembled by asymmetric bis‐urea motifs to balance self‐healing performance and high toughness.^[37]^ However, the design of such heterogeneous system with higher *T*_*g*_ values would limit the ion migration,^[38]^ resulting in decreased ionic conductivity.^[39]^ More importantly, this heterogeneous system frequently lacks the control of mobile ion dynamics, which often generate a high initial capacitance based on the iontronic sensing mechanism, thereby creating a poor sensitivity.[^38^, ^40^] Thus, it remains a substantial challenge to confer superior self‐healing, extreme toughness, and effective ion dynamics to an iontronic material.

Herein, we proposed a molecularly engineered strategy of introducing carboxyl‐functionalized groups into “dynamic hard domains” of poly(urethane‐urea) (PUU) matrix, achieving a piezo‐ionic elastomer (CPIE) with high fracture toughness, extreme crack resistance and autonomous self‐healing. In such a system, the sequential rupture of hierarchical hydrogen bonds with different binding strengths within dynamic hard domains effectively dissipate energy during deformation, leading to super fracture toughness. Meanwhile, the rapid rearrangement of dynamic hydrogen bonds provides dynamic hard domains with low binding energy and high mobility, allowing autonomous room‐temperature self‐healing (Figure 1b). Moreover, the inclusion of (1‐butyl‐3‐methylimidazole chloride ([EMIM]^+^[Cl]^‐^) (IL) launched a piezo‐ionic mechanism, exhibiting mechanically sensitive ion capture and release features due to the electrostatic interactions between carboxyl groups and ion pairs (Figure 1b). Specifically, the carboxyl groups serve as ion exchange sites, allowing ions to quickly pass through the dynamic hard domains under external pressure. This enables the capture and release of ions under pressure (UP), imparting the sensitive piezoelectric ion dynamics. The extraordinary combination of high toughness, autonomous self‐healing and mechanosensitive ion dynamics allows CPIE to play a more central role in the pressure‐induced electronic skin (e‐skin) applications.

## 2 RESULTS AND DISCUSSION

### 2.1 Biomimetic design and fabrication strategy of CPIE

Human skins are tough, crack‐resistant, and self‐healable materials composed of ion‐rich, graded yet repairable structures, characterized by hard collagen fibrils interwoven into a soft elastin matrix.[^41^, ^42^] These two phases are not only capable of self‐healing with the help of blood cells and platelets but also endow high fracture toughness to skin by resisting the crack propagation through collagen fibrils (Figure 1a). Both characteristics ensure the robustness of the skin to increase service life. Additionally, the unique functional characteristics of mechanoreceptors, which transmit external mechanical stimuli through bound ion channels, ensure that human skin has ultra‐sensitive pressure sensing ability across a broad spectrum of pressures.[^43^, ^44^] Under pre‐stimulus conditions, the phospholipid bilayer on the cell membrane acts as a gate, closing the protein channel to prevent the transduction of sodium ions. However, upon external stimulation, compression leads to the opening of the ion channel, inducing the transport of Na^+^ ions to the generation of chemical signals, which are subsequently converted into neuronal signals and ultimately transmitted to the central brain, resulting in the sensation of touch (Figure 1a).

Inspired by the remarkable features of human skin depicted above, we present a conceptual design of CPIE to mimic the high toughness, exceptional self‐healing and controllable ion dynamics in human skin (Figure 1b). Specifically, we first designed a kind of PUU with carboxylate ion units incorporated in its hard segments (denoted as CE, Figures S1–S3). The alicyclic structures of hexamethylene diisocyanate and isophorone diamine (IPDA) in the hard segments of CE created significant steric hindrance, which helped prevent crystallization, leading to the supramolecular assembly of distinct “dynamic hard domains” structures conducive to high toughness and autonomous self‐healing of CE (Figure 1c). Subsequently, the CPIE was achieved after blending CE with IL, in which the [EMIM]^+^[Cl]^‐^ pair was steadily trapped to the carboxylate anions on CE main chains through electrostatic interactions in the static state, preventing the leakage of IL as well (Figure 1b). However, such electrostatic interactions would be broken to release free cations and anions as the CPIE was compressed with pressure, which moved oppositely to the different surface to form electric double‐layers (EDLs), thus generating a high variation in capacitance to dramatically improve the sensor sensitivity (Figure 1b). Upon removal of external pressure, the free ions could be recaptured rapidly again. This mechanosensitive ion trap and release characteristic through electrostatic interactions is the core of piezo‐ionic mechanism in this system, which is akin to the rebalancing of potential via ionic movement through protein channels in human skin.

### 2.2 Optimization of autonomous self‐healing and fracture toughness of CE

**FIGURE 2:**
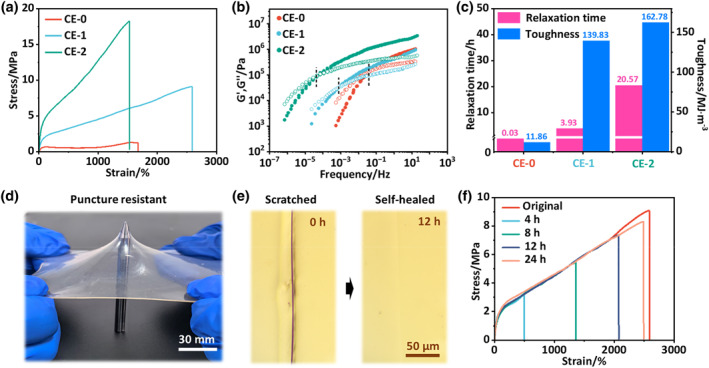
Optimization of autonomous self‐healing and fracture toughness of CE. (a) Stress‐strain curves of CE‐x (*x* = 0, 1, 2) with different contents of IPDA motifs. (b) Master curves of the frequency dependence of G′ and G″ for CE‐x (*x* = 0, 1, 2) at a reference temperature of 25°C. (c) Relaxation time and fracture toughness of CE‐x (*x* = 0, 1, 2). (d) Photographs demonstrating the puncture resistance of CE‐1 film with a 0.8 mm thickness. (e) Optical microscopic images of the scratch‐healing process of CE‐1 at 25°C. (f) Typical stress‐strain curves of the pristine and healed CE‐1 for various healing time intervals at 25°C.

To establish the optimum conditions for efficient autonomous self‐healing and high fracture toughness in the CPIE, various CEs (denoted as CE‐x, *x* represents the molar weight of IPDA in CE, Table S1) were synthesized with a stepwise increase of the molar ratio of IPDA in the hard segments of PUU matrix, aiming at the fabrication of an optimal hard domains structure simultaneously possessing high binding strength and fast dynamics to a great extent. As shown in the stress‐strain curves of all CEs (Figure 2a), due to the absence of IPDA chain extenders that could introduce a large number of irregularly arranged urea H‐bonds in the hard segments, CE‐0 exhibited the worst mechanical properties with a tensile strength of 1.29 MPa, a tensile strain of 1675%, and a low toughness of 11.86 MJ m^−3^ (Figure 2a and Table S2). In contrast, CE‐x with strengthened hard domains by IPDA motifs showed an excellent combination of remarkably improved tensile strength, strain and toughness, which is highly desired but normally difficult to realize. With the molar ratio of IPDA increasing from 1 to 2 mmol, the tensile strength and toughness was gradually enhanced while the stretchability decreased concomitantly. Specifically, the tensile strength of CE‐x increased from 9.08 to 18.22 MPa, the tensile strain decreased from 2577% to 1527%, and the toughness increased from 139.83 to 162.78 MJ m^−3^, which was approximately 12.7 times that of CE‐0 (Figure 2c). These mechanical results demonstrated that the implantation of the IPDA motif results in the dynamic reinforcement for hard domains, ensuring the high strength and toughness of the corresponding CE‐x elastomers.

Subsequently, rheological experiments were conducted to evaluate the dynamic viscoelasticity of CE‐x, in which the reference temperature was set at 25°C (self‐healing temperature) based on the time‐temperature superposition principle.[^45^, ^46^] As depicted in Figure 2b, completely different relaxation behaviors were found depending on the contents of IPDA motifs, where the storage modulus (G′) and loss modulus (G″) exhibited distinct crossover frequency points (*ω*_term_), corresponding to the terminal relaxation behavior of CE‐x. It is possible to estimate that the feature relaxation times (*τ*_term_, calculated using the 1/*ω*_term_) were 0.03 h, 3.93 h, 20.57 h for CE‐0, CE‐1, and CE‐2, respectively (Figure 2b). Substantially, *τ*_term_ was closely related to the required time to achieve healing at the reference temperature. With the decrease of *τ*_term_, the reconfiguration dynamics of the self‐repairing polymer network become faster.[^16^, ^47^] Considering the experimental results of dynamic relaxation time and mechanical toughness (Figure 2c), the optimal sample was CE‐1 with the best combination of fast dynamics and high toughness. As a proof of concept to demonstrate its high toughness, CE‐1 with a thickness of 0.8 mm could easily tolerate puncture from a sharp needle with maintained structural integrity (Figure 2d).

To visualize the self‐healing process, scratch tests were then conducted on CE‐1 at ambient temperature (25°C, relative humidity (RH): 50%), where a visible artificial scratch faded away after autonomous healing for 12 h (Figure 2e), demonstrating excellent scratch recovery capability. Resplicing tests were subsequently carried out to quantify the self‐healing efficiency of CE‐1. Specifically, the sample film was cut into two pieces, and then the fracture surfaces were brought into contact for self‐healing. Figure 2f illustrates the typical tensile curves of the original and reconnected specimens for various healing durations at a tensile speed of 100 mm min^−1^. Self‐healing efficiency (*η*) was defined as the recovery of toughness calculated from integral areas under stress‐strain curves.^[48]^ As expected, the healing efficiency of CE‐1 gradually increased with healing time, which was up to a high value of 93.33% upon the healing time being extended to 24 h (Figure 2f). Such a high healing efficiency is comparable to many reported self‐healing polyurethane and polyurea,[^37^, ^49^, ^50^, ^51^] polyurea,[37,49–51]which is due to the rapid reformation of the dissociated hydrogen bonds within dynamic hard domains.

### 2.3 Optimization of comprehensive performances for CPIE

**FIGURE 3:**
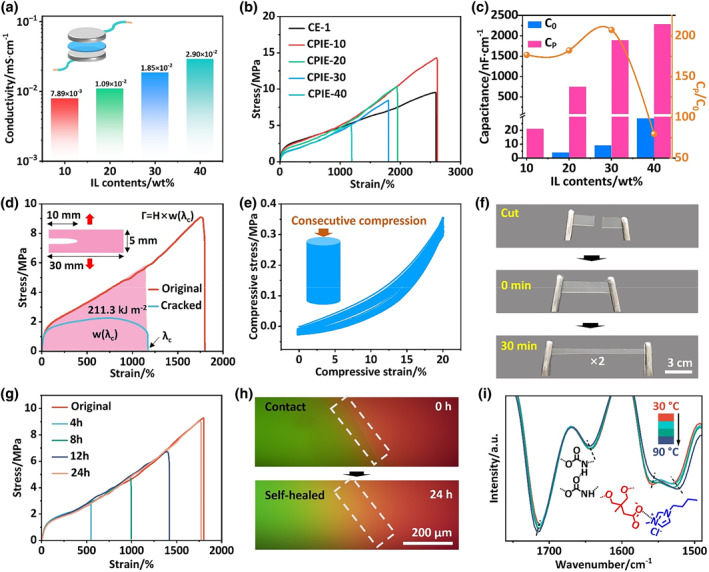
Comprehensive performances for CPIE. (a) Ionic conductivity of CPIE‐x with various IL contents ranging from 10 wt% to 40 wt%. (b) Tensile stress‐strain curves of CPIE‐X. (c) Pressure‐dependent capacitance changes of CPIE‐x. (d) Stress‐strain curves of CPIE‐30 intact specimens and precut crack specimens (gauge length: 5 mm). The fracture energy (*Γ*) is calculated by the formula *Г* = *H* × *W(λ*_*c*_ *).* (e) Consecutive cyclic loading‐unloading curves of CPIE‐30 with a compressive strain of 20%. (f) Photographs of CPIE‐30 self‐healing instantly at 25°C. (g) Stress‐strain curves of the original and self‐healed CPIE‐30 at 25°C for different healing times. (h) Fluorescence microscopic images confirming the chain diffusion process of CPIE‐30. (i) Temperature‐dependent FT‐IR spectra of CPIE‐30 at heating of 25–90°C.

CPIE were prepared with the optimized CE (i.e., CE‐1) as a matrix and different contents of [EMIM]^+^[Cl]^–^ IL. Four kinds of CPIE‐x (*x* denotes the loading percentage of IL) were fabricated with the increase in the IL content from 10 to 40 wt%. Figure 3a shows the ionic conductivity of CPIE‐x as a function of IL contents. As depicted, the conductivity steadily increased with the IL contents, which was due to the presence of mobile ions with high contents. Subsequently, the mechanical properties of CPIE‐x were characterized from the stress‐strain curve. Upon introducing 10 wt% ILs, the tensile stress of the obtained CPIE‐10 increased compared to that of CE‐1 (Figure 3b). This improvement was due to the formation of hydrogen bonds between the hydrogen atoms in [EMIM]^+^ and the carbonyl oxygen in CPIE, which could act as the reversible crosslinking points to enhance the mechanical strength of CPIE‐10.[^52^, ^53^] However, as the ILs contents were further increased, the mechanical properties including stress, strain and toughness of CPIE‐x (*x* = 20, 30, 40) decreased correspondingly due to the plasticization effect of the ILs within CPIE matrix (Figure 3b).[^8^, ^32^] Nevertheless, the CPIE‐x still retains excellent mechanical properties after incorporating a large number of ILs, Typically, the tensile strength and toughness of CPIE‐30 with 30 wt% ILs were up to 9.28 MPa and 83.43 MJ m^−3^ respectively (Table S2), much higher than many reported ionic gels or elastomers.[^1^, ^33^, ^54^] As for piezo‐ionic mechanism presented for tactile sensing, the variation of capacitance UP is the decisive factor for high device sensitivity. Taking this into account, we investigated the pressure‐dependent capacitance changes of CPIE‐x, in which the initial (*C*_*0*_) and final capacitance (*C*_*p*_) values were recorded as a function of IL contents under external mechanical pressure (Figure 3c, Table S3). As depicted, the *C*_*0*_ and *C*_*p*_ were positively correlated with the ionic conductivity, that is, the higher the ionic conductivity was, the higher the initial and final capacitances were.[^7^, ^15^, ^55^] However, the rate of capacitance variation (*C*_*p*_/*C*_*0*_) showed different trends with the increase of IL contents. CPIE‐30 possessed the highest *C*_*p*_/*C*_*0*_, corresponding a maximum output sensitivity accordingly (Figure 3c), and as a result, the optimal IL content for CPIE was 30 wt% (CPIE‐30), which also had high conductivity and mechanical toughness. The details of CPIE‐30 possessing the highest capacitance variation rate and the related mechanism are discussed in the Figure S5.

The optimal CPIE‐30 ionic elastomer exhibited extreme crack resistance due to its unique dynamic hard domain structure (Figure S4), which could redirect existing cracks and prevent further propagation according to the Cook‐Gordan crack arrest mechanism (Figure 1b).^[56]^ The fracture energy was used to quantitatively measure the cracking resistance of materials by the widely used Rivlin‐Tomas pure shear method.^[37]^ Figure 3d shows the stress‐strain curves of the crack and intact CPIE‐30 specimens. In the CPIE‐30 specimen with a width of 30 mm and a crack of 10 mm, no crack growth occurred even when stretched to 11.73 times its initial length (5 mm). The corresponding calculated fracture energy was up to 211.27 kJ m^−2^. Such an extremely high fracture energy value is over 123.54 times that of tough human skin (1.71 kJ m^−2^),^[41]^ and even surpasses those of Al and Zn alloys (0.60–190.27 kJ m^−2^).^[41]^ According to our previous works, we considered this superior fracture energy of CPIE‐30 was attributed to the dynamic hard domains assembled by graded hydrogen bonds, resisting the crack propagation to alleviate local stress concentration.^[37]^ Besides, the optimal CPIE‐30 also had excellent elasticity, which was highly desired for piezoelectric sensory applications. Consecutive pressure loading‐unloading experiment was employed to evaluate the elasticity at 20% strain with a compressive rate of 10 mm min^−1^. As shown in Figure 3e, all the loading and unloading curves of CPIE‐30 almost overlapped with a negligible lag and residual strain in 20 consecutive cycles, demonstrating its excellent elastic restorability. In addition, the optimal CPIE‐30 ionic elastomer exhibits a high transmittance of up to 90% at visible wavelengths at room temperature (Figure S6).

Owing to the high room‐temperature dynamics of CE‐1 matrix that dominated by optimized hard domains and molecular structure, the derived CPIE‐30 ionic elastomer exhibited autonomous and effective self‐healing ability in the absence of external stimuli. As depicted in Figure 3f, the complete cut scenario of two individual CPIE‐30 specimens could be healed together to sustain deformation for 30 min at ambient temperature (25°C, RH = 35%). Furthermore, as confirmed by the tensile stress‐strain curves (Figure 3g), the mechanical properties of CPIE‐30 fully recovered after a 24 h healing period, showing a significantly high self‐healing efficiency of 96.40%. Noting that this value was slightly higher than that of CE‐1 (93.33%), which might be due to the plasticization of the IL that promotes the migration of polymer chains across the fracture interface. To determine the autonomous room‐temperature self‐healing mechanism, we subsequently employed fluorescence microscopy to monitor the local interpenetration of the interfacial macromolecules across the damaged interfaces at room temperature. As shown in Figure 3h, a clear color fusion which overlapped red with green luminescence distinctly emerged after contact for 2 h at 25°C, confirming the chain mobility diffusion contributing to the self‐healing process of CPIE‐30. Besides, the reversible breakage‐reformation of the dynamic supramolecular interactions also facilitates the self‐healing of CPIE‐30 at the molecular scale. Such multiple hydrogen bonds and electrostatic interactions within CPIE‐30 matrix were characterized by temperature‐dependent FT‐IR (Figure 3i). As the temperature increased from 25 to 90°C, the peak at 1712 cm^−1^ from the hydrogen‐bonded C=O moiety in the urethane and urea groups, and the peak at 1643 cm^−1^ from the hydrogen‐bonded N‐H moiety in the urethane and urea groups shifted to higher wavenumbers^[26]^; meanwhile, the C=O moiety at 1527 cm^−1^ from the carboxyl groups and the C=N moiety at 1562 cm^−1^ from [EMIM]^+^ moved to lower wavenumbers.[^1^, ^11^, ^55^] These variations elucidated the breakage of hydrogen bonds and electrostatic interactions upon heating, generating free motifs accordingly.

### 2.4 Characterization of molecular interactions and piezo‐ionic dynamic mechanism

In this mechanosensory system, the core of piezo‐ionic dynamic mechanism of CPIE is the electrostatic interactions between [EMIM]^+^ cation in ILs and carboxylate anion groups in CE polymer chains, which not only improved the compatibility of ILs within CE matrix, but also initiated the trap and release phenomenon. Therefore, we first confirmed such an interaction through the contrastive analysis of internal molecular structures of ILs, CE, and CPIE characterized via attenuated total reflection Fourier‐transform infrated spectroscopy (Figure S7). As shown in Figure S5, the characteristic spectral bands of C=N and C‐N asymmetrically stretched at 1570 and 1164 cm^−1^ in [EMIM]^+^ shifted to 1562 and 1161 cm^−1^, respectively.[^15^, ^57^] These variations suggested that the Coulomb interaction between IL pairs was weakened due to the [EMIM]^+^ cation being dragged away in CPIE by carboxylic acid anions. To further demonstrate the trapping effect of [EMIM]^+^ on the ‐COO^‐^ group, we designed a control sample without carboxyl groups (denoted as PU‐IL‐30, Figure S1), which contained 30 wt% ILs as well. In a sharp contrast, there were no shifts of C=N and C‐N asymmetric stretching in the [EMIM]^+^ in PU‐IL‐30 (Figure S7), which demonstrated the crucial role of ‐COO^‐^ group to trap [EMIM]^+^ through electrostatic interaction.

**FIGURE 4:**
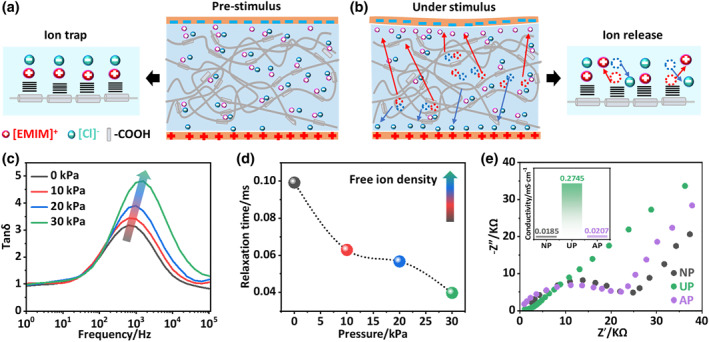
Piezo‐ionic dynamic mechanism and working principle of CPIE device based on trap and release. Design of CPIE‐30‐based piezo‐capacitive device consists of CPIE‐30 film sandwiched between two deformable Ag NW/CE‐1 electrodes. (a) [EMIM]^+^[Cl]^‐^ ion pairs are trapped to ‐COO^‐^ groups under a pre‐stimulus condition. (b) [EMIM]^+^[Cl]^‐^ ion pairs are released under pressure stimulus due to the pressure‐mediated rapture of electrostatic interaction based on ion pumping effect. (c) Ion dynamics and the concentration of free ions under increased pressure. (d) Changes in charge relaxation time with pressure in CPIE‐30. (e) Nyquist plots of CPIE‐30 under no pressure (NP), under pressure (UP), and after pressure removal (AP). Insert showed the corresponding conductivity under each condition. Ag NWs, Ag nanowires.

After molecular characterization, we proceed to investigate the piezoelectric capacitance pressure sensing mechanism (known as mechanically sensitive piezoelectric ion dynamics) through fabrication of CPIE‐30‐based devices sandwiched between two deformable Ag nanowires (Ag NWs)/CE‐1 self‐healing electrodes (which includes a penetrating network of Ag nanowires on the CE‐1 surface), facilitating the formation of an electrical double layer at the interface (Figure 4a). Under static conditions, most of [EMIM]^+^[Cl]^‐^ ion pairs are captured and confined by ‐COO^‐^ groups via electrostatic interaction. Nonetheless, under external stimuli, CPIE‐30‐based device started to deform progressively, thereby shortening the distance between the upper and lower electrodes and creating a powerful electric field (Figure 4b). Notably, the disruption of electrostatic interactions due to deformation and pressure causes the ion pairs to detach from the ‐COO^‐^ groups, creating the mechanically sensitive piezoelectric ion dynamics.[^58^, ^59^] Owing to this efficient ion‐pumping ability, the established ion trap and release phenomenon prior to and following the induced pressure provided low initial capacitance and high final capacitance value, respectively. As such, the precise control of piezo‐ionic dynamics was achieved, resulting in highly improved device sensitivity and high signal‐to‐noise level. Noting that the smaller [Cl]^–^ ions in CPIE were more mobile than the larger [EMIM]^+^ ions UP.^[60]^ The difference in diffusion rates was established throughout the film, forming a more pronounced EDL,^[61]^ which increased sensitivity to pressure variation and enhanced piezo‐ionic sensitivity by ensuring effective control of piezo‐ionic dynamics.^[62]^

To demonstrate the above notion of ion trap and release, we studied the correlation between piezoelectric ion dynamics and alterations in complex impedance behavior by performing stress relaxation experiments on both CPIE‐30 and PU‐IL‐30 reference samples (without the ‐COO^‐^ group) under applied pressure. As the pressure was applied on CPIE‐30‐based device, its impedance gradually decreased as a function of increased pressure (Figure S8a), attributed to the increase in ions that can move freely when pressure was applied. In a sharp contrast, the impedance of the PU‐IL‐30 (without ‐COO^‐^ groups)‐based device exhibited no substantial variation under applied pressure (Figure S8b). This is because no ions were trapped at rest, and as a result, no pressure (NP)‐induced ion pumping occurred UP (Figure 4a). Moreover, we employed the frequency dependence of tanδ (*Ɛ*”/*Ɛ*′) to study the number density and diffusion rate of free ions by analyzing the displacement of the relaxation peak.[63]The time scale of ion motion is called the charge relaxation time (*τ*), and the frequency at which intersection between the imaginary impedance and real impedance in Bode's diagram is called the charge relaxation frequency (1/*τ*).^[63]^ As shown in Figure 4c, the 1/τ shifts to a higher frequency as the applied pressure increases, which was a shift not observed in PU‐IL‐30 (Figure S8c). This is because the increase in the concentration of freely moving ions leads to an acceleration of ion relaxation, resulting in a gradual decrease in relaxation time with increasing pressure (Figure 4d).[^64^, ^65^, ^66^] Finally, electrochemical impedance spectroscopy Nyquist plots were used to characterize the reversible ion movement of CPIE‐30 under pressure (UP), after pressure relief (AP), and without pressure (NP). The results in Figure 4e clearly proved the reversible recovery of ion movement after removing pressure, whereas no significant reversible ion motion occurs in the PU‐IL‐30 curve (Figure S7) because most of the ion pairs were in the polymer matrix.^[15]^

### 2.5 Piozocapacitive tactile sensing performance

**FIGURE 5:**
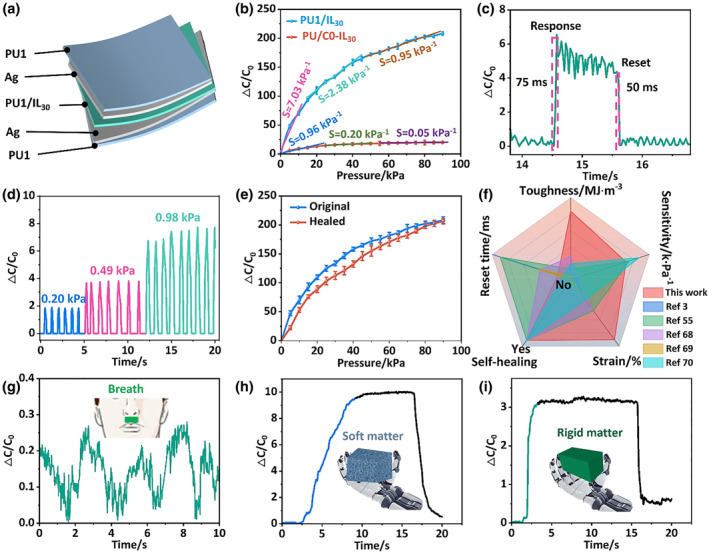
Capacitive sensing performance and practical application of the CPIE device. (a) Schematic diagram of a piezocapacitive sensor based on a CPIE‐30 iontronic material. (b) Pressure sensitivity (100 mv applied bias at 1 kHz) comparison between CPIE‐30 and PU‐IL‐30 (reference sample). (c) Trend of capacitance changes over time when different pressures (0.20, 0.49 and 0.98 kPa) were applied. (d) Instantaneous response time of the CPIE‐30‐based sensor under a 700 g (0.686 kPa). (e) Original and self‐healing CPIE‐30 film under static pressure conditions. (f) Comprehensive comparison between the CPIE‐30‐based sensor and related self‐healing ion sensors regarding toughness, sensitivity, response time, self‐healing capability, and fracture energy. (g) Detection of respiratory changes by attaching the sensor on the philtrum. The sensors respectively monitor the capacitive signals on objects of varying hardness grabbed by the robotic arm: (h) soft foam and (i) rigid spectacle case grabbed by the robot hand.

With the CPIE‐30 as the dielectric layer, we fabricated an e‐skin demo, and its specific structure is shown in Figure 5a. Ag nanowires were employed as electrodes, which were embedded in the critical surface of CE‐1 surface to ensure the deformability of the electrodes. Noting that CE‐1 and CPIE‐30 had the same polymer composition, which could be integrated together to realized self‐healing of the whole sensor architecture. The pressure‐sensing performances of the CPIE‐30‐based device were investigated under bias voltages of 100 mV at 1 kHz. The pressure sensitivity (Figure 5b), defined as *S* = *δ*(*ΔC*/*C*_*0*_)/*δP* (in which *C*_*0*_ is the initial capacitance, *ΔC* is the change of capacitance, and *P* is the applied pressure),^[67]^ of the CPIE‐30‐based device was 7.03, and 0.95 kPa^−1^ during 0–15, 15–55, 55–90 kPa, respectively. PU‐IL‐30 was employed as dielectric layer to assemble a piozocapacitive sensor was used for comparison. It can be seen that the pressure sensitivity of the PU‐IL‐30‐based device at each stage (0.96, 0.20, 0.05 kPa^−1^) was substantially much lower than that of CPIE‐30 (Figure 5b). Moreover, the sensitivity of PU‐IL‐30 over 30 kPa represented a typical saturation behavior, while CPIE‐30 showed extraordinary sensitivity over the same range. Such a high‐pressure sensitivity of CPIE‐30‐based device was due to ‐COO^‐^ group‐induced piezo‐ionic dynamics, leading to high *C*_*p*_/*C*_*0*_ values. Upon increasing pressure, changes over time were observed through dynamic response and recovery (Figure 5c). The capacitance signal of the CPIE‐30‐based device demonstrated high reliability and durability with excellent repeatability. To directly measure tactile perception capability, the finger tough was pressurized on CPIE‐30‐based device. Visible signals appeared upon a finger tough, further proving the high sensitivity (Figure S10).

Since the electrostatic interaction between ILs and ‐COO^‐^ groups in CPIE‐30 was highly sensitive to external force, the response time of CPIE‐30‐based sensor was greatly shortened. Specifically, as a ∼0.70 kPa pressure was loaded, the response times were 75 and 50 ms during loading and release, respectively (Figure 5d), showing negligible delay. Additionally, the whole CPIE‐30‐based sensor possessed excellent autonomous self‐healing performance, and therefore, it could repair damage or cuts even when the sensitivity was slightly reduced (Figure 5e). This autonomous self‐healing process, especially the spontaneous fusion of Ag NW percolation network, was attributed to the self‐healing driving force of CPIE‐30 connecting the crack interfaces. To demonstrate the advantages of our CPIE‐30‐based sensor, this work was compared to the representative iontronic pressure sensors (Figure 5f, Table S4), which simultaneously showed remarkable characteristics, including higher toughness (83.43 MJ m^−3^), superior sensitivity (7.03 kPa^−1^) and responding time (50 ms), extremer fracture energy (211.27 kJ m^−2^) and highly effective self‐healing (96.40%).[^3^, ^55^, ^68^, ^69^, ^70^]

To illustrate the potential of our sensors in real life applications, we performed several representative functional demos. First, CPIE‐30‐based device was employed to monitor the slight changes in airflow during the respiratory process. As depicted in Figure 5g, clear respiratory signals were obtained based on the relevant capacitance variation. This demonstrates the potential application of this piezocapacitive device in detecting abnormal respiratory rates in medical settings. Second, we utilized the tactile sensing performance to identify soft and hard objects. The sensors were attached to foam and glasses cases respectively to identify different hard objects. The robotic arm was controlled by the system, and the inductance capacitance resistancre meter recorded changes in capacitance in real time. Since foam is prone to deformation during grasping, the pressure received by the robotic arm gradually increased, and finally the pressure tended to a steady state. This phenomenon was reflected in the capacitance signal as a slow rise, followed by the appearance of a plateau where the capacitance reached its maximum value and then stabilized (Figure 5h and Movie S1). In contrast, the spectacle case was less prone to deformation during grasping. As a result, the pressure exerted by the robotic hand instantaneously reached its maximum value, and the capacitance signal promptly reached its highest value (Figure 5i and Movie S2). Accordingly, these illustrate provides strong evidence that the device has great potential for medical diagnosis and can facilitate human‐machine interaction, owing to its remarkable mechanosensitivity.

## 3 CONCLUSION

In this work, by mimicking the ion‐rich, graded yet repairable structure of human skin, we demonstrate a tough, crack‐resistant and autonomous self‐healing CPIE material with mechanosensitive piezo‐ionic dynamics based on a molecularly engineered strategy, that is, introducing carboxyl‐functionalized groups into hard segment of self‐healing poly(urethane‐urea) to assemble a new generation of dynamic hard domains. This structure gives the piezo‐ion skin advantages characteristic of biological skin, including high toughness (83.43 MJ m^−3^), superior fracture energy (211.27 kJ m^−2^), autonomous self‐healing (96.40% efficiency) and effective control of ion dynamics. Notably, a high pressure sensitivity of 7.03 kPa^−1^ is realized for this piezo‐ionic skin, which is ascribed to the reversible electrostatic interaction from ‐COO^‐^ groups triggering the ion pumping process in CPIE. This extraordinary combination of properties indicates that the CPIE‐based piezo‐ionic sensor will play a more pivotal role in emerging wearable electronics and smarter human–machine interfaces, in which the robustness of devices highly relies on the fracture toughness, crack resistance and self‐healing performance of the materials.

## CONFLICT OF INTEREST STATEMENT

The authors declare no competing interests.

## ETHICS STATEMENT

No animal or human experiments were involved in this study.

## Supporting information

Supporting Information S1

## Data Availability

All relevant data supporting the results of this study are available in the article and its supplementary information files. Further data are available from the corresponding authors upon request.
