# Supplementary material for: Highly tough, crack‐resistant and self‐healable piezo‐ionic skin enabled by dynamic hard domains with mechanosensitive ionic channel
Source: Smart Mol. 2024 Aug 19;2(3):e20240008. doi: 10.1002/smo.20240008 (PMC12118253; doi:10.1002/smo.20240008)
Supplement: Supplementary file 1 — Supporting Information S1 [file SMO2-2-e20240008-s001.docx]

Supporting Information

**Highly** **tough, crack-resistant** **and self-healable piezo-ionic skin enabled by dynamic hard domains with mechanosensitive ionic channel**

XueBin Wang^1^, Tong Liu^1^, FuYao Sun^1^, Jingyi Zhang^1^, BoWen Yao^1^, JianHua Xu^1,*^, and JiaJun Fu^1,*^

^1^ School of Chemistry and Chemical Engineering, Nanjing University of Science and Technology, Nanjing 210094, China.

.

**Experimental Section**

**Materials**

Dicyclohexylmethylmethane-4 (HMDI, 99%), was purchased from Adamas. Polytetramethylene ether glycol (PTMEG, average M_n_=~2,000), Dimethylolbutanoic acid (DMBA), Isophorone diamine (IPDA), 3,3-Bis(hydroxymethyl)pentane (DMPT), 1-butyl-3-methylimidazole chloride ([EMIM]^+^[Cl]^-^) were supplied by Aladdin Reagent, China. Acetone and Chloroform were purchased from Sinopharm Group Chemical reagent Co., LTD. Acetone was dried with molecular sieves for 2 week before use. Other chemicals used were analytical reagents without further purification.

**Synthesis of CE elastomer**

PTMEG (5 g, 2.5 mmol) and DMBA (0.444 g, 3 mmol) were dissolved in a 50 mL round bottom flask with 5 ml acetone as solvent, and then stirred at 80 °C for 10 min. Subsequently, the HMDI (1.73 g, 6.6 mmol) dissolved in 10 ml acetone was slowly added into the above solution. After that, the reaction mixture was continuously stirred at 80 °C for 3 h, then cooled to 55 °C and added to IPDA (0.17g, 1mmol) for another 7 h. Then, triethylamine (2mmol) was dropped into the flask at 55 °C and reacted for 10 min. Finally, CE-1 emulsion was obtained by intense emulsification indeionized water (20 mL) for at least 1 h. The whole synthesis process was under condensation reflux and nitrogen atmosphere. The obtained product was poured into the polytetrafluoroethylene (PTEE) mold and dried at room temperature for 48 h. The synthesis process of CE-0, CE-2 elastomers were similar to that of CE-1, except that the molar ratio of IPDA is changed (Table S1).

**Synthesis of PU control elastomer**

PTMEG (5 g, 2.5 mmol) and DMPT (0.396, 3 mmol) were dissolved in a 50 mL round bottom flask with 5 ml acetone as solvent, and then stirred at 80 °C for 10 min. Subsequently, the HMDI (1.73 g, 6.6 mmol) dissolved in 10 ml acetone was slowly added into the above solution. After that, the reaction mixture was continuously stirred at 80 °C for 3 h, then cooled to 55 °C and added to IPDA (0.17g, 1mmol) for another 7 h. The whole synthesis process is under condensation reflux and nitrogen atmosphere. Finally, the obtained product is poured into the polytetrafluoroethylene (PTEE) mold and dried at room temperature for 48 h.

**Fabrication of** **CPIE iontronic elastomers**

The specific preparation process of CPIE-30 was as follows: Initially, 300 mg mL^-1^ IL (5 mL) was poured into 350 mg mL^-1^ CE-1 emulsion (10 mL) under quickly stirring for about 12 h. Subsequently, the mixture was transferred into a mold and allowed to dry at 50°C for 48 h. Finally, the dried CPIE-30 iontronic elastomer was obtained. A series of ionic elastomers, CPIE-10, CPIE-20, and CPIE-40 (that is CE/IL-x ,where x represents the percentage of IL in the total mass of CE-1 and IL, with values of 10 wt%, 20 wt%, and 40 wt0% respectively), were prepared using the same method.

**Fabrication of PU-IL-30 control sample**

PU and IL were dissolved in chloroform at a mass ratio of 7:3, followed by continuous stirring for 12 h. Subsequently, the mixture was transferred into a mold and allowed to dry at room temperature for 48 h. Finally, the dried PU-IL-30 ionic elastomer was obtained.

**Fabrication of self-healing piezocapacitive sensor.**

The piezocapacitive sensor consists of a dielectric layer between the top and bottom conducting layers and encapsulation layers (Figure 5a). Initially, CPIE-30 was completely dissolved and then poured into a Teflon mold. After drying, silver paste was coated onto the surface of the CPIE-30 the conducting layer. Finally, smooth CE-1 thin films (used as the encapsulation layer) were covered onto the electrodes to create a piezocapacitive sensor with an ideal total thickness of approximately 1.2 mm. The piezocapacitive sensor using PU-IL-30 as the dielectric layer and PU as the encapsulation layer is manufactured through the same procedures.

**Characterizations**

The ^1^H NMR spectrum was recorded using a Bruker AVANCE III 500 MHz spectrometer at room temperature with deuterated chloroform as the solvent. The FTIR spectrum was recorded in the range of 4000 cm^-1^ to 600 cm^-1^ using a Bruker Tensor II spectrometer equipped with a Specac Golden Gate MK II ATR accessory. The sample was pressed into the Specac Golden Gate MK II ATR attachment for FTIR measurements that vary with temperature, with a temperature gradient ranging from 25°C to 90°C at intervals of 20°C. Fluorescence microscopy images were recorded with a Jiangnan BM2000 fluorescence microscope and the sample were stained with Rhodamine B and Fluorescein, and the samples containing different stains were combined after cutting and observed with the fluorescence microscope. The assembled piezocapacitive sensor was measured using the precision Agilent 4294A Impedance Analyzer.

**Mechanical Test**

The tensile tests were conducted on a Shimadzu AGS-X testing machine. Unless otherwise specified, all tensile tests were carried out at room temperature (25 °C) with a strain rate of 100 mm min^-1^. The dimensions of the tensile test specimens were 10 mm gauge length × 5 mm width × 0.6-0.8 mm thickness. Toughness is defined as the integral area under the stress-strain curve. For cyclic compression tests, loading and unloading processes were performed on the AGS-X testing machine at a strain rate of 10 mm min^-1^ at 25 °C. Rheological behavior was evaluated on a TA AG R2 rheometer (20 mm diameter parallel steel plate). Frequency scanning was performed with a 0.1% strain amplitude in the frequency range 0.1 to 100 rad s^−1^. The rheological master curve was constructed using the TTS principle, and the temperature was regulated by Peltier plate (25-105 °C).

For fracture energy tests, pure shear tests were performed with 500 N load cell. This method required two sets of samples with the same size of the width of 30 mm, the thickness of 0.7 ± 0.2 mm, and the gauge height of 5 mm. One set of samples were precut with a crack of 10 mm width, the other set was without crack. The fracture energy value (Γ) is calculated by the formula:

$$\text{Г=H}\times\text{w}\left(\text{λc}\right)$$

**Electrochemical Measurements**

The Electrochemical impedance spectroscopy (EIS) test on CPIE-30 was conducted using the CHI 760E electrochemical workstation within the frequency range of 0.1 Hz to 100 kHz. the ionic conductivity of the symmetric stainless steel/CPIE-30/stainless steel cell was calculated using the following formula:

$$\sigma\text{=}\frac{\text{L}}{\text{R}\times\text{A}}$$

where L and A are the thickness and area of CPIE-30 membrane, respectively, and R represents the bulk electrolyte resistance.

**Self-Healing Testing**

For mechanical properties of self-healing test, each sample was cut into two completely independent parts using blade. Subsequently, the cut surfaces were gently brought into contact without applying any pressure and self-healed at 25°C. Then, the healed samples were subjected to tensile testing at 25°C with a strain rate of 100 mm·min^-1^. The healing efficiency *η* is calculated as the ratio of the integrated area under the stress-strain curve of the fully healed specimen to that of the original specimen. Measurements were carried out on three or more samples at each healing time to obtain an average value.

**Supporting Figures**


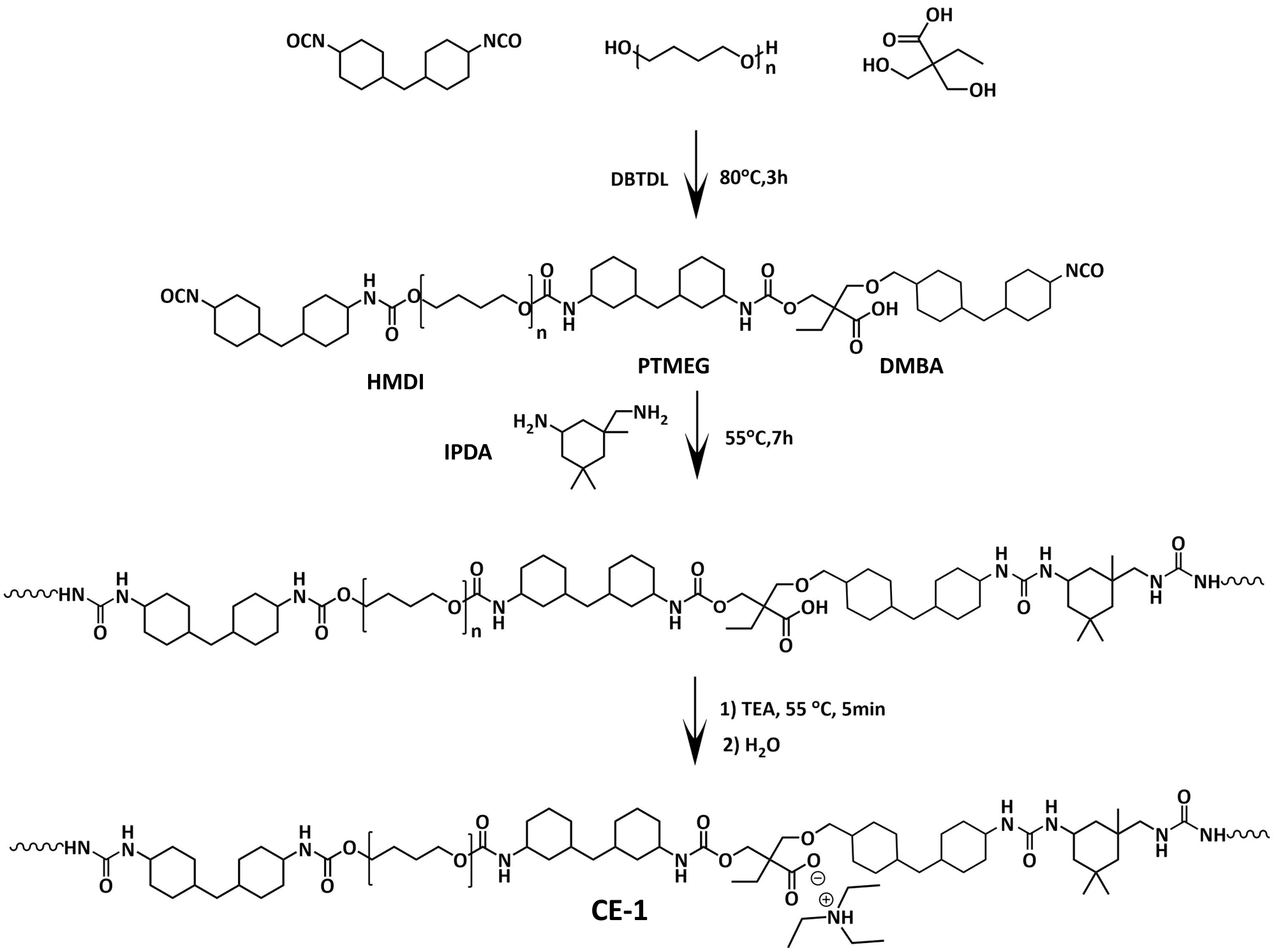


**Figure S1. Schematic illustration of the synthetic procedure of CE-x**


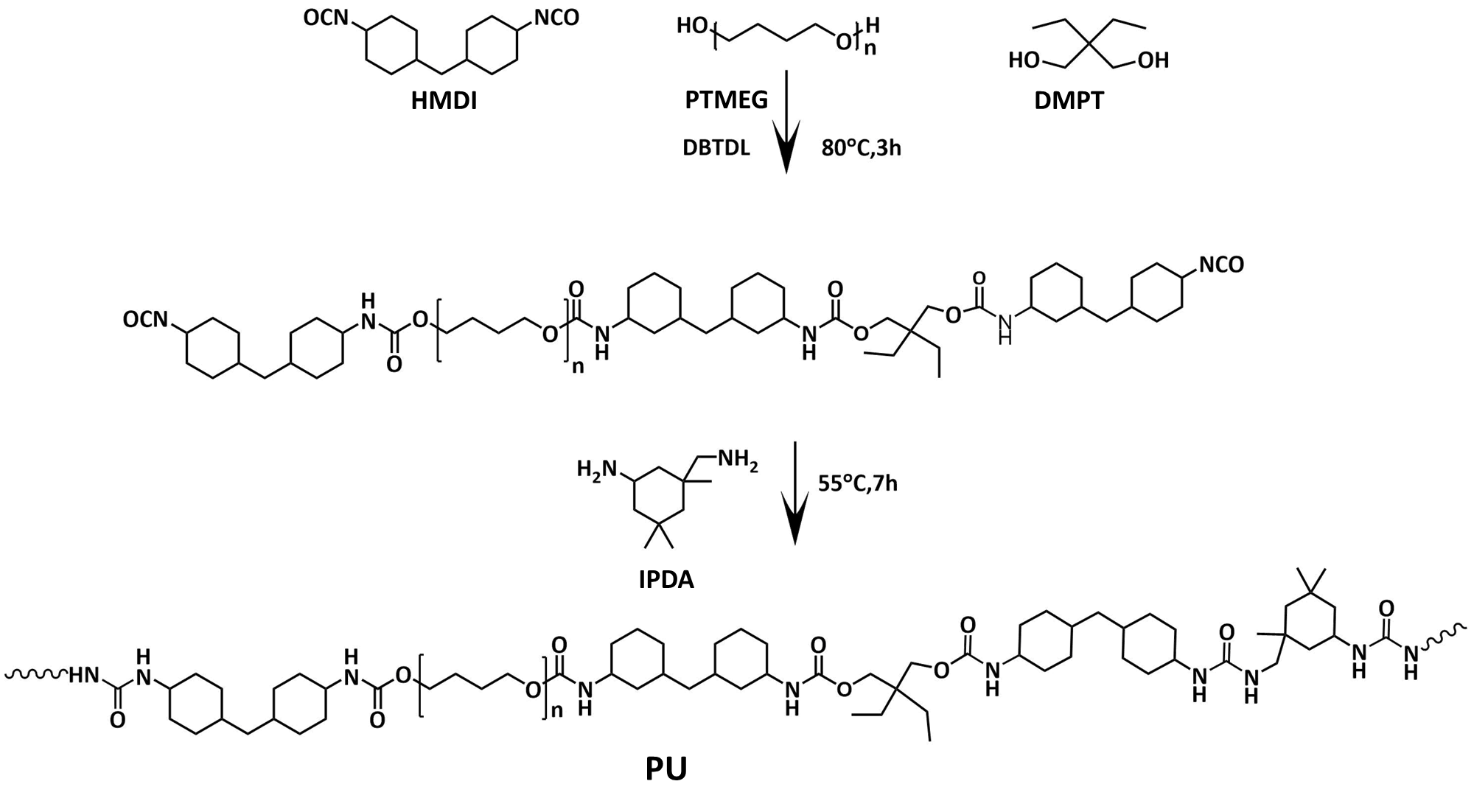


**Figure S2. Synthesis method of PU control sample without COOH groups**


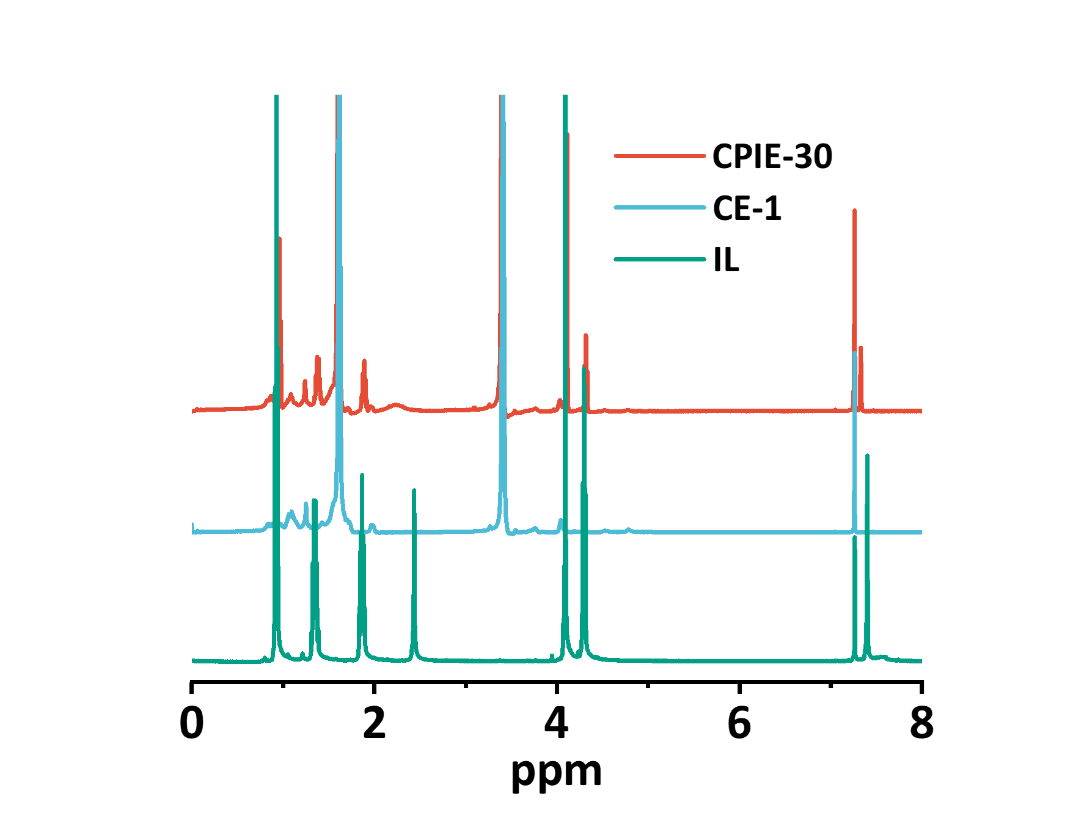


**Figure S3. ^1^H NMR spectra (500 MHz, CHCl_3_, 298 K) of ILs、CE-1、CPIE-30**. The identification of NMR characteristic peaks and the corresponding molecular structure. 0.91/2.11 ppm (-CH3, HMDI, IPDA), 1.09/4.03 ppm (-COOH, DMBA), 1.60/3.4 ppm (-CH_3_, PTMG), 6.82/7.01 ppm (-NH-CO-NH-) and 2.46 ppm (H_2_O, from atmosphere).


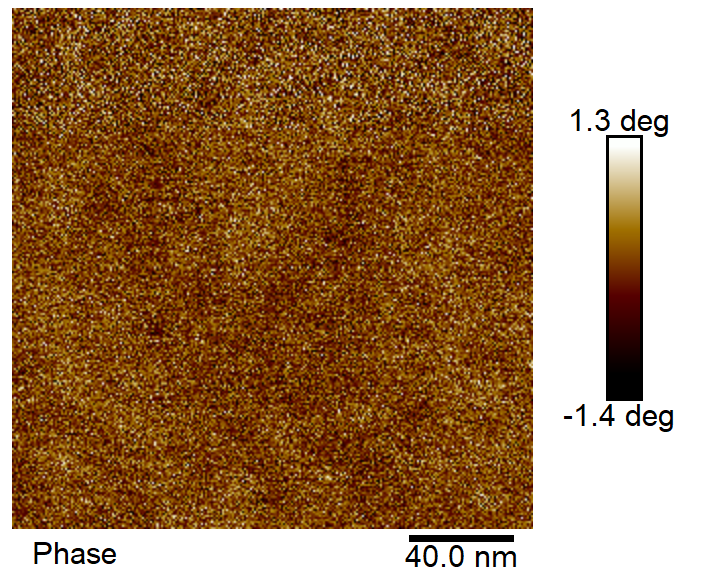


**Figure S4. AFM images of CE-1 elastomer.** The dark region represents the soft phase and the bright region represents the hard phase, respectively, demonstrating the distinct phase separation structure of CE-1.


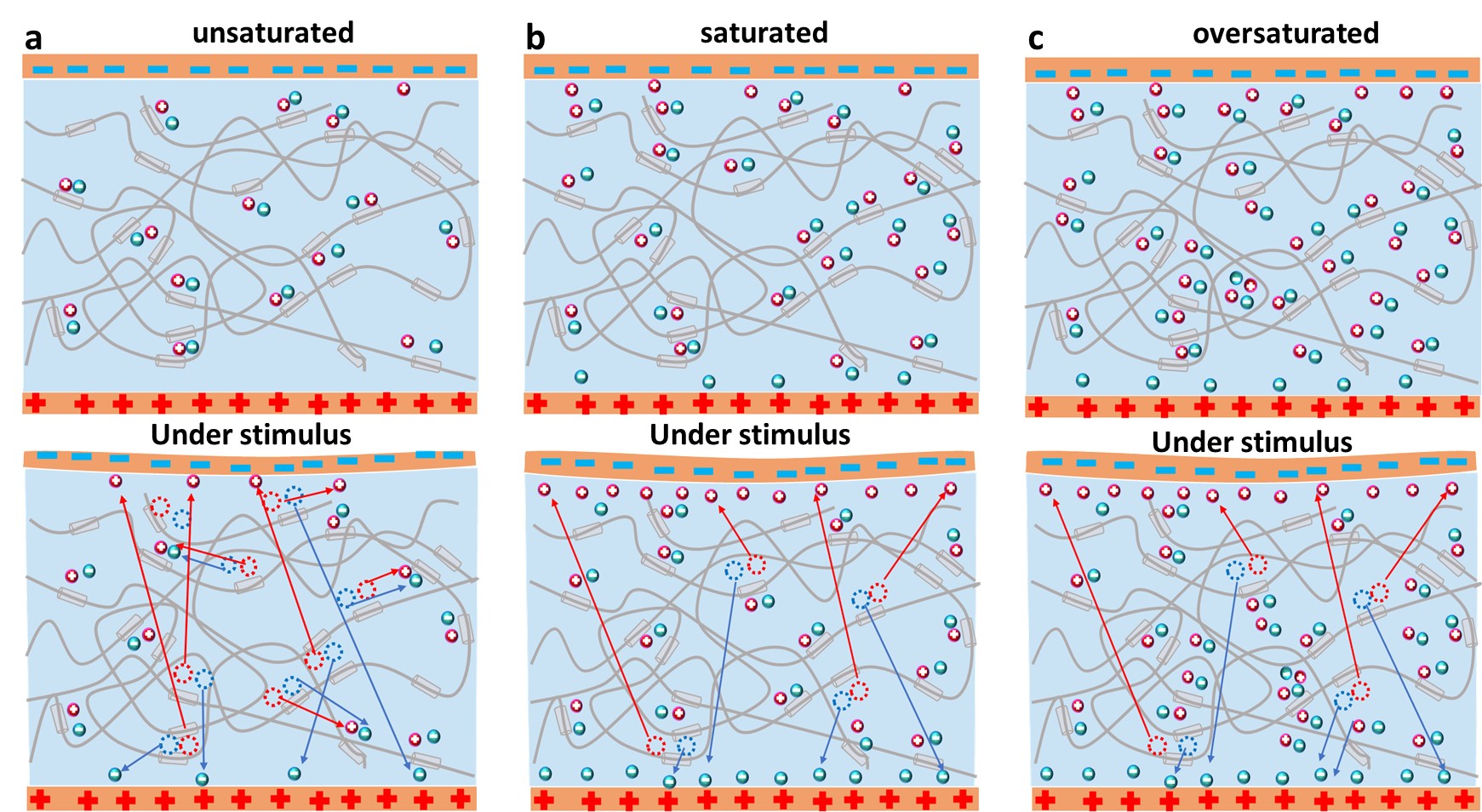


**Figure S5.** **Molecular interactions and piezo-ionic dynamic mechanism.** The piezo-ionic dynamic mechanism and working principle of CPIE-x (a) with a small amount of IL, (b) with a saturated IL and (c) with an excess amount of IL.

CPIE-30 possessed the highest capacitance variation rate.

When the binding sites of carboxyl groups were unsaturated, the ions in CPIE-10 and CPIE-20 could move quickly under pressure. This movement caused some ILs relocating from the pressure center to the periphery and band to the binding sites. Consequently, IL was redistributed so that the capacitance change rate was smaller.

In CPIE-30, the saturation of the binding site of ions leads to the intensive accumulation of IL in the double electric layer to form effective charge under pressure, and a large capacitance change rate was obtained. However, in CPIE-40, the IL was oversaturated, causing excess [EMIM]^+^[Cl]^-^ ions to form large ionic clusters within the polymer, creating numerous local charge regions. This led to a sharp increase in *C_0_*, while the excess ions did not exhibit a pressure-mediated ion pumping effect, resulting in a less significant increase in *C_p_*. Consequently, C*_0_* increases faster than C*_p_*, which indicates that *C_p_/C_0_* values of CPIE-40 are lower than those of CPIE-30. As a result, the CPIE-30 has the highest capacitance change rate.


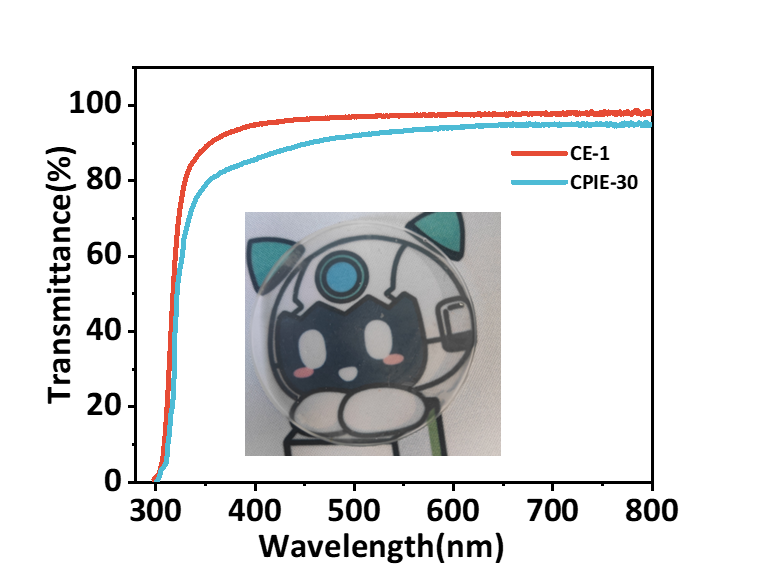


**Figure S6. UV-vis spectra of** **CE-1 and CPIE-30.**


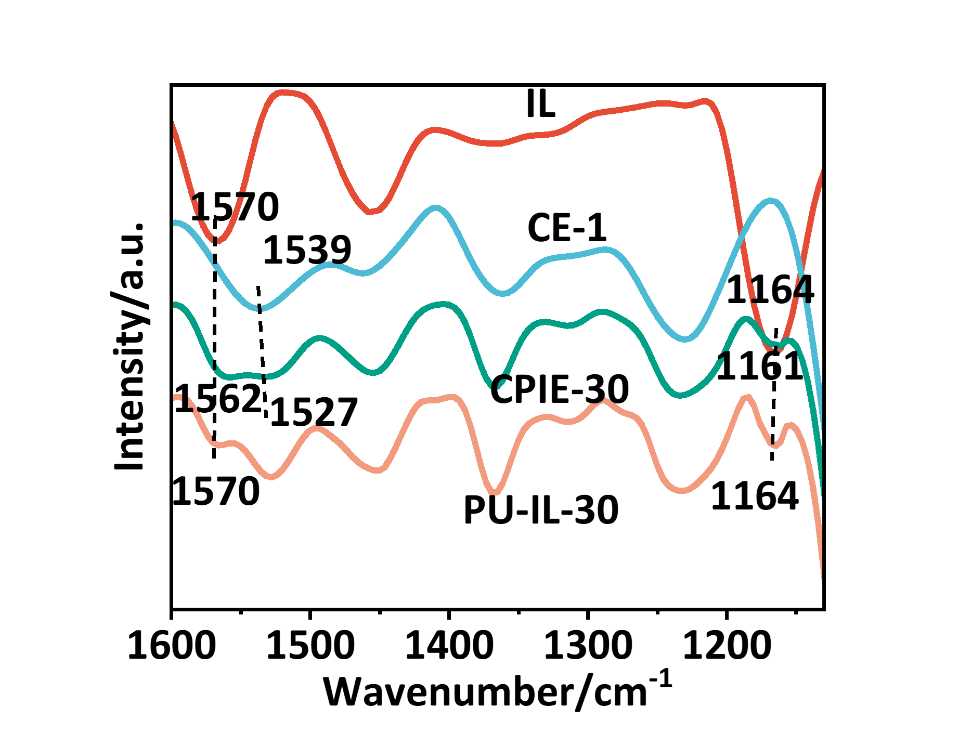

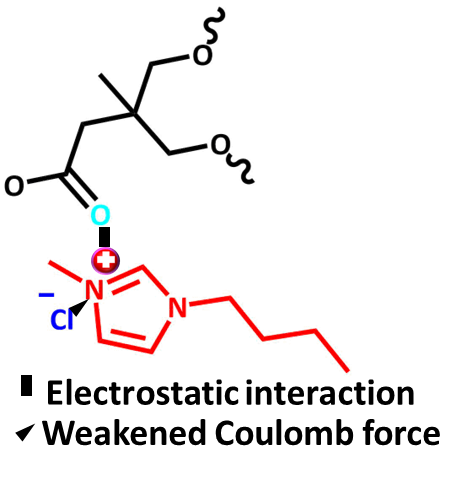


**Figure S7. FT-IR spectra of IL, CE-1, CPIE-30, and PU-IL-30 control sample.**


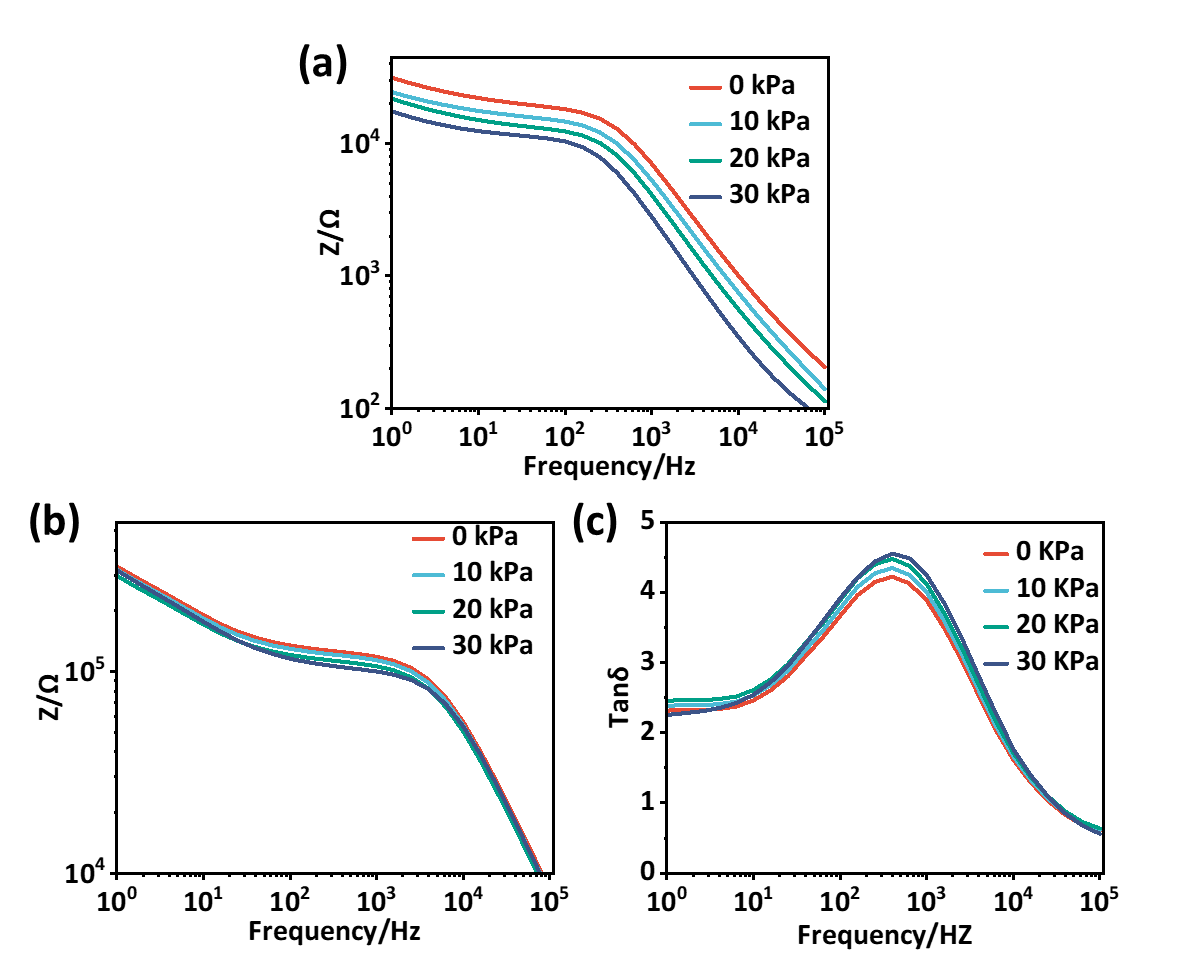


**Figure S8. Ion trap and release effect. Relationship between piezo-ionic dynamics and the changes in complex impedance behavior of (a) CPIE-30 and (b) PU-IL-30 as a function of applied external pressure. (c) Ion dynamics and free ion concentration of PU-IL-30 with a stepwise pressure increase.**


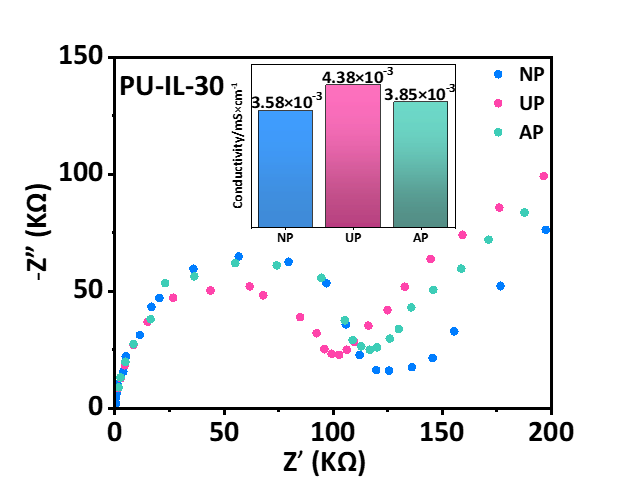


**Figure S9. Impedance Nyquist diagram of PU-IL-30. Ionic conductivity of PU-IL-30 under no pressure (NP), with pressure (UP), and without pressure (AP) (insert displays ionic conductivity under each condition).**


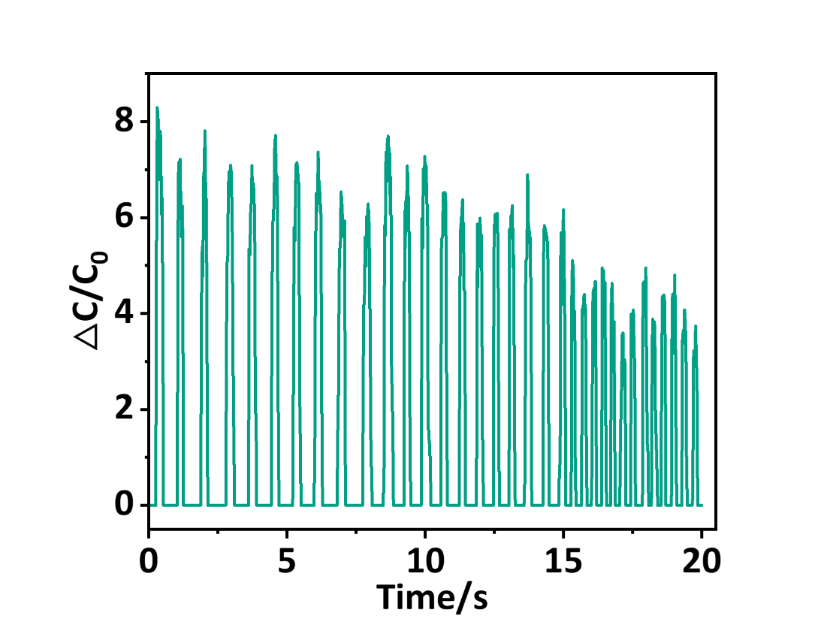


**Figure S10. Capacitive signals of the CPIE-30-based sensor by finger pressing.** When the finger tapping frequency increases, the capacitance change increases accordingly.

**Supporting Tables**

**Table S1.** Content values of the main monomers in the synthesis of CEs

| samples | PTMEG/g | HMDI/g | DMBA/g | IPDA/g | Molar ratio  PTMEG : HMDI : DMBA : IPDA |
| --- | --- | --- | --- | --- | --- |
| CE-0 | 5 | 1.2 | 0.444 | 0 | 2.5 : 5.6 : 3 : 0 |
| CE-1 | **5** | **1.47** | **0.444** | **0.17** | **2.5 : 6.6 : 3 :1** |
| CE-2 | 5 | 1.73 | 0.444 | 0.34 | 2.5 : 7.6 : 3 : 2 |

**Table S2.** Summary of mechanical properties of CE-0, CE-1, CE-2, PU elastomer and CPIE-10, CPIE-20, CPIE-30, CPIE-40, PU-IL-30 iontronic materials

| Sample | Tensile strength  MPa | Strain  % | Toughness  MJ m^-3^ |
| --- | --- | --- | --- |
| CE-0 | 1.29 | 1675 | 11.86 |
| CE-1 | **9.08** | **2590** | **139.86** |
| CE-2 | 18.22 | 1527 | 162.78 |
| CPIE-10 | 16.04 | 2614 | 210.01 |
| CPIE-20 | 11.35 | 1950 | 110.48 |
| CPIE-30 | **9.28** | **1802** | **83.43** |
| CPIE-40 | 5.60 | 1193 | 37.84 |
| PU | 8.67 | 2006 | 84.51 |
| PU-IL-30 | 3.10 | 1163 | 27.93 |

**Table S3**. CPIE with varying IL concentration (10-40 wt%) presenting the initial and final capacitance values under pressure range of 0~90 kPa to approach saturation.

| **Sample** | ***C_0_***  **nF*cm^-2^** | ***C_p_***  **nF*cm^-2^** | ***C_p_/C_0_*** |
| --- | --- | --- | --- |
| CPIE-10 | 0.12 | 15.62 | 130.66 |
| CPIE-20 | 4.13 | 73.45 | 182.13 |
| CPIE-30 | **9.05** | **1893.71** | **207.23** |
| CPIE-40 | 28.70 | 2284.36 | 79.69 |

**Table S4**. Comparison of self-healing efficiency, toughness, fracture energy, response time, and sensitivity with previously reported iontronic pressure sensors.

| Sample | Toughness /MJ m^-3^ | Tensile strength /MPa | Fracture energy /kJ m^-2^ | \| **Self-healed** \| \| --- \| | \| **Response time**  **/ms** \| \| --- \| | Ref. |
| --- | --- | --- | --- | --- | --- | --- | --- | --- |
| \| ICPS \| \| --- \| | 1.01 | 0.74 | / | No | 300 | \| Ref. 3 \| \| --- \| |
| PANI@WPU | 12.2 | 7.5 | / | Yes | 270 | Ref. 55 |
| PSeD-U20-12h | 4.5 | 0.16 | / | Yes | 125 | Ref. 68 |
| PANI@WPU | 62.3 | 14.2 | / | No | 117 | Ref. 69 |
| I-Skin-i | 19.4 | 8.7 | / | Yes | 32 | Ref. 70 |
| CPIE-30 | **83.43** | **7.05** | **211.3** | **Yes** | **50** | **This work** |
